# Determinants of willingness to pay for the retreatment of insecticide treated mosquito nets in rural area of eastern Ethiopia

**DOI:** 10.1186/s12939-015-0249-9

**Published:** 2015-10-24

**Authors:** Sibhatu Biadgilign, Ayalu Aklilu Reda, Haji Kedir

**Affiliations:** Independent Public Health Research Consultants, P.O. Box 24414, Addis Ababa, Ethiopia; Department of Public Health, College of Health Sciences, Haramaya University, P.O. Box 1014 Harar, Ethiopia; Global Health, Brown Advanced Research Institutes (BIARI), Brown University, Providence, Rhode Island USA; Addis Continental Institute of Public Health, P.O. Box 1014, Addis Ababa, Ethiopia; Department of Sociology, Brown University, Providence, Rhode Island USA

**Keywords:** Willingness to pay, Retreatment, ITN, Rural, Ethiopia

## Abstract

**Objective:**

Is to determine willingness to pay(WTP)/demand for the retreatment of Insecticide treated mosquito nets for malaria control in Ethiopia.

**Methods:**

A community based cross-sectional study was conducted in Gursum district in Eastern Ethiopia. A total of 335 households were surveyed using a pre-tested structured questionnaire. Multivariable regression analyses using the Tobit model were used to test the theoretical validity of elicited WTP.

**Results:**

About 159(76.4 %) of them have received a treated insecticide when they obtained. One hundred twenty five (60.4 %) know that the net should be retreated. Around 110(50.7 %), 80(36.9 %) and 27(12.4 %) of the participants feel that the current price of ITNs as negotiable/ not as such expensive, expensive and cheap. About 306(96.5 %) of them reported that they support that ITNs be given freely and 257(82.9 %) were mentioned that the retreatment service should be provided without charge. The WTP amounts ranged from 0 to 10.4 USD. The mean with SD of the respondents from open ended elicitation method for willingness to pay was 1 USD and 1.53 USD. The reduced tobit regression models showed that average income more than 10.4 USD per month and those household who live within a distance in 30 min to the health facility were the determinant for willingness to pay.

**Conclusion:**

The mean with SD of the respondents for willingness to pay was 1 USD and 1.53 USD. Average monthly income and those household who live within a distance in 30 min to the health facility were determinant for willingness to pay. Government and other development partners should seek a mechanism to make a subsidy or free of charge for the retreatment services. Differential treatment from the public to address the poor and vulnerable households and those who are living far distance from the local health facility is warranted.

## Background

According to WHO, there were an estimated 219 million cases of malaria (range 154–289 million) and 660 000 deaths (range 610 000–971 000) in 2010 [1]. Insecticide-treated mosquito nets (ITNs) have been shown to be an effective and cost-effective means for the control of malaria, especially among children under 5 years [2]. The use of insecticide treated nets is effective in reducing all cause malaria mortality and morbidity between 17 and 43 % in children under 5 years and provides protection to pregnant women who are most susceptible to malaria. ITNs (Insecticide Treated Nets) are easy to use and require less technical and capital outlay to implement compared with other vector control methods. They are cost-effective, which has led to widespread implementation of ITNs by countries on a large scale [3].

Access to bed nets or their delivery, acceptability and compliance with net use are the other critical issues in the success of any ITNs programme. Aside from access to mosquito nets and insecticides, one of the problems in the large-scale implementation of the ITNs programme is re-treatment of the net [4]. ITN use has however been limited due to the cost outlay households require to make towards the purchase of nets, households’ inability to associate the effectiveness of the net with the insecticide leading to low re-treatment rates in most settings and the seasonality associated with the spread of malaria [3].

In Ethiopia, almost 68 % of the 73 million populations in the country live in malarious areas covering almost 75 % of the land. The strategy for selective vector control measure was ITNs distribution based on segmented market approach and through health facilities and campaigns with prioritizing high risk areas and population group. The target for ITNs is to achieve distribution of 2 ITNs per household (on average) in 90 % of the ITNs targeted areas by 2007 [5]. The determination of consumer preferences and demand for different vector control strategies becomes pertinent, when viewed against the background of community involvement as part and parcel of vector control tools and as consumers are expected to contribute some money for the financial sustainability of the delivery of the strategies. Consumer preferences should also guide resource allocation decisions so that people preferences and potential demand for the different tools are satisfied [6].

Several studies documented for the willingness to pay for the ITN in the country. In a baseline survey in four regions of Ethiopia, SNNPR, Tigray, Amhara and Oromia, the main reasons cited being lack of money to purchase it and affordability is one of the determinant factors that impede the possession and use of ITNs. As to their maximum WTP, 47 % suggested 10 Birr or less, 11 % said 11–20 Birr, 28 % from 21 to 50 Birr [7], in Western Shoa Zone, shows that, 99 % did not have prior experience of using bed net but only 4 % of them were unwilling to pay for ITN. The main reasons for unwillingness were inability to afford and lack of confidence in the bed net [8] and 850 (86.5 %) were willing to buy an ITN (if supplied by the market) in Arba Minch area, the common reasons for their unwillingness-to-buy were inability to afford and believed that they do not have to buy it since some people are getting ITNs free of charge [9]. Many studies have provided estimates of mean willingness to pay (WTP) for malaria prevention and treatment [10]. But studies on WTP for malaria control interventions have been uncommon [11]. Therefore, this study is aimed to determine willingness to pay/demand for the retreatment of Insecticide treated mosquito nets for malaria control in Ethiopia.

## Methods

### Study setting and context

This cross-sectional community based study was conducted in Gursum district, Eastern Harerghe zone of the Oromia region in January 2008. Gursum district with a total area of 76,261 ha with a latitude between 907′ and 9032′ N, at longitude of 42017′ to 42038′҆E, and an altitude between 1200 and 2950 m above sea level coupled with the annual average rainfall is 450–600 mm. Gursum has a unimodal rainfall pattern ranging from June to August and the minimum and maximum temperatures are 15 and 35 °C, respectively [12]. The source population included all households with children under 5 years of age. Households were randomly selected from a list provided by the district administration. Health extension workers (HEW) distributed the Long lasting insecticide-treated net (LLIN) to the community at their vicinity/households in a procedure in the form of free mass distribution campaigns. The local malaria control program provide and distribute ITN with the support from the district Health office. The volunteer community workers and local administration conduct social mobilization and raising awareness in the district. ITN distribution is carried out after provision of health education and promotion to the district community. Retreatment was done by the local malaria expert in the area. The study was conducted in Janauary 2008.

### Measurements

To assess the people’s willingness-to-pay/demand, contingent valuation method using binary with follow-up was used through household survey mechanisms. Hence, the contingent valuation method is a valid and justifiable tool for determining peoples’ valuation of goods and services and tested in ITN [13]. The contingent valuation method (CVM) is a survey-based approach for eliciting consumer’s monetary valuations for programme benefits for use in cost-benefit analysis (CBA) [14]. WTP is the maximum a person or household would be willing to pay for a good or service and is one route for providing an estimate of benefit for use in CBA [15].

It uses an artificial market to measure consumer preferences by directly asking their willingness to pay or willingness to accept for change in the level of good or service as well capture the total value of the good- both use and non-use values and its flexibility facilitate valuation of a wide range of non-market goods. We use direct open-ended format- it is one of the way of describing for uncovering values simply by asking the respondent what maximum price he/she is willing to pay for the given retreatment of ITN/ described good in one question. This method has the advantage of avoiding starting point biases. But it is characterized by large number of non-responses and protest zero [16]. WTP for ITN retreatment was the dependent variable. The independent were socio-demographic, economic variables, malaria related questions.

### Questionnaire and data collection

Data were collected using a pre-tested structured questionnaire prepared in English and then translated to Oromiffa, the local language of the area. The questionnaire was adopted from instruments developed by the WHO and UNICEF [17]. Pretest was carried out on 5 % of the households. Necessary modifications were made thereafter. Trained interviewers administered the questionnaire through house to house visits. Information was collected from the heads of the household (father or mother) whenever possible, or from an adult household member in case this was not possible. The study investigators supervised the data collection process.

### Statistical analysis

Data were entered, cleaned using SPSS and analyzed on STATA 12 version. Descriptive summaries (frequencies and proportions) were calculated. Multivariable regression analyses using the Tobit model were used to test the theoretical validity of elicited WTP. Tobit model was used as many of the respondents stated zero WTP amounts [18], adjusted odds ratios (AOR) and their corresponding 95 % confidence intervals (CI) were used to examine the strength of association with the main variable of interests. P-values of less or equal to 0.05 were considered significant.

Ethical approval was obtained from Institutional Research Ethics Review Committee of Haramaya University, Faculty of Health Sciences. A supporting letter was obtained from the district health office after explaining the purpose and significance of the study. Verbal consent was obtained from individual respondents.

## Results

### Socio-demographic characteristics of the study population

A total of 335 households participated in this study. The mean (SD) family size of the households was 5.45 (2.85) with a median of 5. The majority (247, 73.7 %) of the respondents were females, while 228 (68.1 %) were illiterate (see Table 1).

**Table 1:** Socio demographic characteristics of participants in Gursum district, Eastern Ethiopia

Variables	Frequency	Percentage
Sex		
Male	88	26.3
Female	247	73.7
Religion		
Christian	51	15.2
Muslim	284	84.8
Marital status		
Single	16	4.8
Married	287	85.7
Divorced	12	3.6
Widowed	16	4.8
Separated	4	1.2
Occupational status		
Farmer	104	31.0
Student	26	7.8
House wife	149	44.5
Government employee	12	3.6
Merchant	39	11.6
others^a^	5	1.5
Educational level		
Illiterate	228	68.1
Read and write only	16	4.8
Primary education (1–6)	40	11.9
Secondary education (7–12)	32	9.6
Above grade 12	19	5.7
Estimated monthly income of the household (USD per month)^b^		
<10.4	133	39.7
10.4–31.14	103	30.7
31.25–51.98	35	10.4
52.08–83.33	38	11.3
>83.33	26	7.8

^a^Others: sales and services, clerk

^b^Exchange rate at the time of the study, 1 USD = 9.6 Ethiopian Birr (ETB)

### Characteristics of retreatment of mosquito nets

**Fig. 1:**
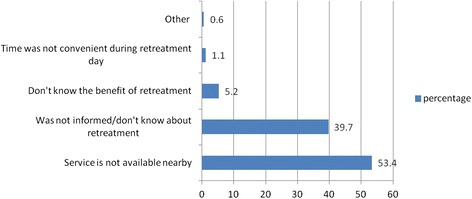
reason mentioned by respondents for not been retreating the insecticide in Gursum district, Eastern Ethiopia

About 159(76.4 %) of them have received a treated insecticide when they obtained. One hundred twenty five (60.4 %) know that the net should be retreated. Around 10 (5.53 %) of the respondents had used ever retreated with an insecticide since its distribution. From this, 6(60 %) and 4(4 %) of them retreated once and twice respectively. The last retreatment date from those who retreated 6(60 %), 1(10 %) and 3(30 %) were <6 months, 6–12 months and >12 months respectively. The retreatment of the net was done by 7 (70 %) health workers at health institution and 3(30 %) self/family member following training. The reason mentioned for the not been retreated with insecticide were 93(53.4 %) service is not available nearby and 69(39.7 %) was not informed/don’t know about retreatment (Fig. 1).

Around 53 (31.93 %) of the respondents mentioned that family member regularly sleep under it for the last one month. About 293(94.82 %) of the participants know the benefit of a mosquito net and 284(95.3 %), 10(3.36 %) and 4(1.34 %) mentioned that it prevents mosquito biting for quite sleep, prevents malaria and prevents the nuisance by other insects respectively. About 185(69.3 %) of them showed that insecticide treated bed net is more effective than untreated one. From this 160(89.45) and 11(6.15 %) were stated that they observe many mosquitoes dead on the floor, bed and the net and no bite at all while sit in home or asleep at bed even without a net. About 123(59.1 %) of the participants never wash the bed net in the 6 months period. About 82(96.5 %) were washed the net with soap and water and 153(80.1 %) know that frequent washing can reduce the efficacy of treated bed net.

### Rating and determinant to willingness to pay for ITN retreatment

Around 110(50.7 %), 80(36.9 %) and 27(12.4 %) of the participants feel that the current price of ITNs as negotiable/not as such expensive, expensive and cheap. About 306(96.5 %) of them reported that they support that ITNs be given freely and 257(82.9 %) were mentioned that the retreatment service should be provided without charge. The WTP amounts ranged from 0 to 10.4 USD. The mean with SD of the respondents from open ended elicitation method for willingness to pay was 1 USD and 1.53 USD [Exchange rate at the time of the study, 1 USD = 9.6 Ethiopian Birr (ETB)].

The LR chi square which measures the overall significance of the model [12.73], i.e., with the null hypothesis that all coefficients are zero is rejected at 1 % significance level showing that at least one of the coefficients is different from zero. The pseudo R2 is 1.24 %, which implies that percentage of the variation in willingness to pay amount is explained by the variables included in the model. Both LR chi square and the pseudo R2 are significant at 1 % implying the model is acceptable to explain the relation between willingness to pay amount and its explanatory variables. Important variables were fitted in model building to assess the determinant of the willingness to pay for the retreatment of ITN. The reduced tobit regression models showed that average income more than 10.4 USD per month and those household who live within a distance in 30 min to the health facility were all statistically significant (*p* < 0.01) for willingness to pay (Table 2).

**Table 2:** Reduced Tobit models for determining the factors that explain WTP for retreatment of ITN in Gursum district, Eastern Ethiopia

Variables	Coefficient	Standard error(SE)	Significant level	Marginal effect	
				prb	intensity
Age	−0.23	0.22	0.29	−0.003	−0.003
Occupation	1.42	1.65	0.391	0.021	0.022
Marital status	0.55	5.84	0.925	0.008	0.1827776
Education	−2.56	5.71	0.654	−0.0396054	−0.8556232
Average Monthly income	3.83	1.89	0.045	0.059234	1.27969
Distance in min to the health facility	8.82	4.43	0.048	0.1363768	2.946
Family size	−0.13	0.22	0.568	−0.00199	−0.043
Know the benefit of a mosquito net	−15.3	12.3	0.216	−0.23719	−5.12
Family member travel anywhere in the last one month	0.81	5.89	0.891	0.124799	0.269
Malaria can lead to death of children	0.49	3.93	0.901	0.00757	0.163
In the past one year, did your family have malaria?	2.89	5.02	0.564	0.0448	0.968
Do you feel your family is at risk of getting malaria	−0.34	4.41	0.939	−0.00526	−0.114
_cons	−8.54	27.36	0.755		

## Discussion

This study aimed at examines the willingness to pay for retreatment for ITN. To assess the demand of the ITN it is better to see how people value the nets and estimated the potential demand for the nets. For this, it is illustrated either examining actual willingness to pay (WTP) as revealed by people’s purchase decisions (revealed preferences), or by determining their stated or hypothetical level of willingness to pay (WTP) for the nets through the contingent valuation method (CVM) [19]. In this study, the demand for ITN purchase was fair and able to obtain freely. This is also supported by studies across the discipline. According to Obinna (2002), stated that communities can possibly pay for the poor to benefit from a community-based insecticide-treated nets (ITNs) programme using the various financing mechanisms that exist at the community level for ITNs and ITNs re-impregnation for those unable to pay for themselves [20].

In another studies, it was documented that hat potential commercial ITN markets will be undermined if free nets are widely distributed, leaving communities with even poorer access once donor funds run out [21] and in Africa the point of to illustrated is to adopt ITNs as a public good—like childhood vaccines—through public sector involvement in highly subsidized or free provision for the vulnerable African lowland rural populations, where the great bulk of the world’s malaria burden is concentrated [22]. In similar way, free insecticide and working with local health workers, more than 90 % of nets are retreated in 2–3 days in Tanzania [23].

In multivariate analysis, income level has a greater contribution with willingness to pay for retreatment of ITN. This is evidenced that in Tanzania high rates of net purchase in towns was observed but much lower rates in rural areas, especially among the people with lowest incomes whose children have the poorest health [22] and reaching the poorest of the poor with malaria control interventions poses great challenges, not solely because of financial barriers to accessing care and prevention services [24]. Previous studies in Ethiopia shows the poor have shown their WTP for reduced cost of ITN [25] and monthly income of the households was not a significant determinant of people’s willingness-to-pay for ITN [9] and as the average monthly income decreased, the WTP for ITN has increased significantly [26]. Along with this, in south western Ethiopia study, there is a close association between respondents economic status and willingness to pay for ITNs retreatment [27].

In our study, distance to the health facility was associated with willingness to pay for the retreatment of ITN. Similar finding has been reported in other studies as well. Non-financial barriers, including distance from health services, and opportunity costs of lost time at work, may also be underlying factors for malaria infection in the poor [24]. In one of Burkina Faso study, distance to health facility negatively influenced WTP, thus longer distance and less WTP [28]. The explanation for this is that those who lived in the rural area cannot get access for the retreatment service and other service package. The poorest populations in developing countries often live in the most remote areas and are socially or culturally marginalized [24].

The study has some limitations. It is difficult to elucidate/disclose the exact income of the household or at individual level and cross-sectional nature of the study for making causality and inference. On top of that, the WTP approach creates a hypothetical market and tries to capture how much they would be willing to pay for the benefits the malaria retreatment provides. Hence, the WTP methods by itself might lead to undervaluation or inflation of the value of the service based on the approach employed and the measure, whether the community understands or imagines the problem, their experience. Though the approach inherently depend on the value that the community gives to the benefit from the service, the findings can be widely used to design policies, prioritize intervention actions, implement cost sharing arrangements etc. As to the knowledge of the authors, this is the first of its kind to determine the WTP for retreatment of ITN services in the country and gives a shade of light for the policy makers to take appropriate decision. There has been discussions on whether public health interventions like malaria net or retreatment can be seen as a purely public good, or marketable good. Given the public importance of these interventions and their abundance in the market at least in many countries, that might not be an easy task to get a best fit. Nonetheless, that doesn’t matter as such in a valuation experiment with a hypothetical setting. And most importantly, an aggregate value by the community for such a service is pertinent in designing policies and strategies in malaria control and treatment. As the study time has passed, we didn’t anticipate what happened recently.

In conclusion, the mean with SD of the respondents for willingness to pay was 1 USD and 1.53 USD. Average income more than 10.4 USD per month and those household who live within a distance in 30 min to the health facility were all statistically significant (*p* < 0.01) for willingness to pay. So the government and other development partners should see the mechanism to make a subsidy or free of charge for the services hence the community can pay more value for the retreatment of ITN since free distribution of ITNs is not a solution unless retreatment services were available to the community to be implemented. Differential treatment from the public to address the poor and vulnerable households and those who are living far distance from the local health facility is warranted and applying other method of evaluation for estimation of willingness to pay may be warranted to probe and synthesize.
